# From Fatima Al-Fihriya to the First Documented Medical Degree in the World

**DOI:** 10.7759/cureus.105954

**Published:** 2026-03-27

**Authors:** Najoua Ghani, Mohamed Taiebine, Moulay Hassan Farih, Abderrahman Tenkoul, Chakib Nejjari

**Affiliations:** 1 Faculty of Medicine, Euromed University of Fez, Fez, MAR; 2 Euromed Research Center, Faculty of Human and Social Sciences, Euromed University of Fez, Fez, MAR; 3 Faculty of Human and Social Sciences, Euromed University of Fez, Fez, MAR; 4 Euromed Research Center, Euromed University of Fez, Fez, MAR

**Keywords:** al-qarawiyyin, education, fatima al-fihriya, gender equity, history, medical degree, women

## Abstract

This paper delves into the history of the University of al-Qarawiyyin, founded in 859 CE in Fez, Morocco, as an important institution in the global history of higher education. Established by Fatima Al-Fihriya, this university marks a significant phase in the Islamic Golden Age, evolving from a mosque-centered religious institution into a multidisciplinary academic center. This review article emphasizes al-Qarawiyyin’s contribution to the preservation and integration of classical Greek and Roman knowledge with Islamic scientific advancements, offering a curriculum that includes theology, mathematics, astronomy, and medicine, among other disciplines. Special emphasis is placed on the university’s pioneering role in the formalization of medical education, highlighting the *Ijaza* as an early model of structured credentialing that serves as a useful parallel to the subsequent development of licensing systems in medieval European universities. While the intellectual contributions of figures such as Ibn Rushd, Ibn Khaldun, and Maimonides spanned the vast landscape of the Islamic world, the University of al-Qarawiyyin served as one important channel through which this knowledge was preserved and transmitted to the West. It functioned as a key node in a wider network of intellectual exchange that linked North Africa, Al-Andalus, the Euro-Mediterranean space, and beyond. This narrative review article highlights the institution's lasting influence on contemporary educational frameworks and the historical significance of female philanthropic leadership in intellectual progress.

## Introduction and background

The period from the eighth to the 13th centuries is often celebrated as the Islamic Golden Age, a time when the Islamic world was at the forefront of scientific, philosophical, and educational progress [1]. This era was characterized by the preservation, enhancement, and dissemination of knowledge, which influenced the intellectual development of both the Islamic world and medieval Europe. Morocco, strategically located at the intersection of Europe and Africa, played a crucial role in this vibrant intellectual environment. This trajectory of institutionalized learning was established upon earlier scholarly traditions that significantly informed the development of later academic centers. For instance, the Academy of Gundishapur (Southwestern Iran around 550 CE), a preeminent center for medical and philosophical inquiry, chronologically predates the founding of the University of al-Qarawiyyin [2]. Similarly, the Medical School of Salerno, although not formally classified as a university, emerged as a seminal center for medical training that antedated the institutional establishment of the University of Bologna, thereby setting a critical precedent for the evolution of higher education in the medieval period [2]. Among its most notable contributions to education is the establishment of the University of al-Qarawiyyin, which is recognized as the oldest degree-granting university in the world [3] by UNESCO and the Guinness World Records, while the University of Bologna in Italy was founded in 1088 and is the oldest one in Europe. Notably, the Sumerians had scribal schools or É-Dub-ba soon after 3500 BC [2]. The founding of al-Qarawiyyin is particularly remarkable because it was established by Fatima al-Fihriya, a woman who leveraged her wealth and influence to promote learning at a time when women's opportunities were limited [1,3].

In this narrative review, we aim to (1) synthesize historical evidence on al‑Qarawiyyin’s role in degree‑granting higher education and early medical credentialing; (2) examine the 1207 Ijaza as one of the earliest documented medical degrees; and (3) highlight Fatima al‑Fihriya’s and other women’s contributions to education and medicine.

The foundation of al-Qarawiyyin University

Fatima al-Fihriya, born in 800 CE in Kairouan (modern-day Tunisia), relocated to Fes following her father's death. Originating from a prosperous and educated family, al-Fihriya inherited considerable wealth, which she dedicated to philanthropic endeavors [4]. Her most significant contribution was the founding of al- Qarawiyyin University in 859 CE, initially conceived as a mosque and later transformed into a center for higher education [1]. Driven by her commitment to advancing knowledge, al-Fihriya’s legacy extends beyond her era, representing the empowerment of women and their essential role in the intellectual and educational advancement of society. The university initially focused on religious studies, particularly Islamic theology, jurisprudence, and the Quran. Over time, it broadened its curriculum to encompass various scientific fields, including astronomy, mathematics, and medicine, drawing scholars from across the Islamic world and beyond [2,4]. Fatima al-Fihriya's establishment not only enriched the intellectual landscape of the Muslim world but also played a crucial role in preserving and transmitting classical knowledge to future generations in both the East and the West [4].

Fatima al-Fihriya is celebrated as a pioneering figure in women's philanthropy and education within the Islamic world. Her contributions extend beyond her role as an educator; she is emblematic of the potential for women to effect significant social change through philanthropy, challenging contemporary gender norms and inspiring future generations [3]. The establishment of the al- Qarawiyyin University was a remarkable act of investment in community welfare, made possible by her inheritance from her father. By prioritizing education over personal luxury, she not only founded a key educational institution but also set a precedent for women's leadership in the pursuit of knowledge and societal advancement [4]. Her legacy has fostered a greater appreciation for women's roles in education, encouraging advocates of gender equality to draw inspiration from her life and achievements.

Scholars at al-Qarawiyyin University engaged with classical Greek and Roman texts, many of which had been translated into Arabic, and integrated this knowledge with Islamic thought to develop new scientific paradigms [2,4]. The university attracted distinguished scholars from various regions of the Muslim world who contributed to its success in advancing scientific and philosophical thought. Al-Qarawiyyin not only served as a hub for intellectuals from Africa and the Middle East but also played a crucial role in the transfer of knowledge to Europe, particularly through the translations of Arabic texts into Latin [2,5,6]. Many of the university's teachings significantly influenced the academic landscape of medieval European universities.

## Review

Sources and methods

This narrative review synthesizes the historical trajectory from the establishment of the University of al-Qarawiyyin by Fatima al-Fihriya in 859 CE to the formalization of early medical certification in the medieval era. Selection criteria for these subjects were strictly predicated on their demonstrable influence on pedagogical institutionalization, prioritizing verified primary manuscript evidence over later hagiographic reconstructions. To resolve competing historical interpretations, the authors employed a comparative critical framework, triangulating established scholarly consensus with extant archival data to reconcile discrepancies and ensure a rigorous, evidence-based synthesis of these foundational academic milestones.

Notable scholars associated with al-Qarawiyyin

**Figure 1:**
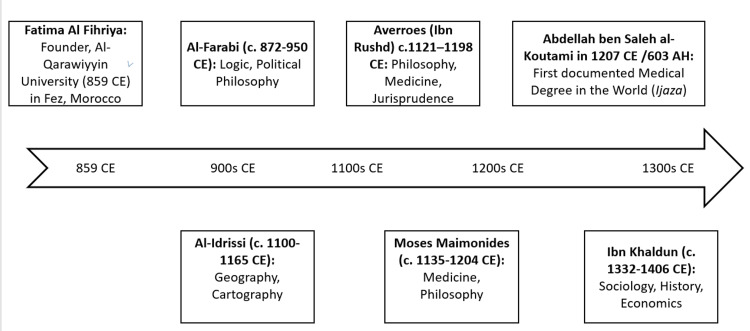
Notable scholars associated with al-Qarawiyyin (8th-14th centuries). Image credit: Mohamed Taiebine. CE, Common Era; AH, Anno Hegirae

The intellectual legacy of al-Qarawiyyin University is characterized by a seamless synthesis of diverse disciplines, where the works of various scholars form a continuous dialogue across centuries (Figure 1).

In the realm of philosophy, the university served as a crucible for reconciling faith and reason. The logical foundations established by al-Farabi, who harmonized Aristotelian and Platonic thought, provided the essential framework for the institution’s curriculum. This rationalist tradition was significantly advanced by Ibn Rushd, whose commentaries on Aristotle bridged Greek logic with Islamic theology, subsequently influencing Moses Maimonides during his tenure in Fez to extend these interdisciplinary methods into Jewish scholasticism [6,7].

The institution’s commitment to empirical inquiry facilitated rapid advancements in geography and medicine. Al-Idrissi revolutionized 12th-century cartography in his 1154 Tabula Rogeriana by integrating diverse geographical data [8], while the medical innovations of Abu Bakr al-Razi, specifically his pioneering studies on infectious diseases and medical ethics in Kitab al-Hawi, were rigorously preserved and utilized for clinical instruction [9].

**Figure 2:**
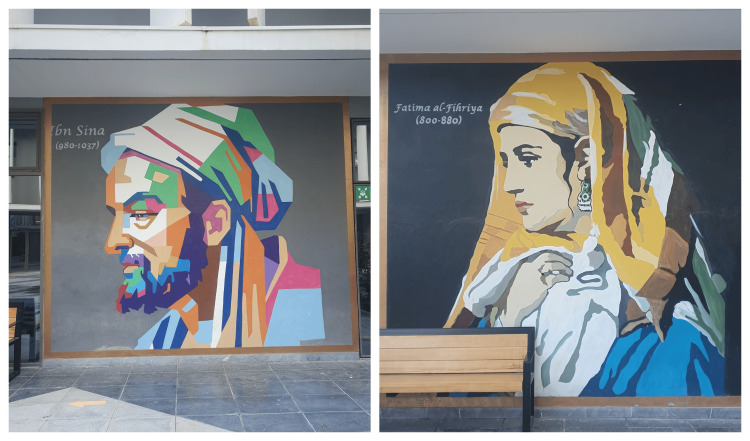
Fatima al-Fihriya and Ibn Sina at the Euromed University of Fez in Morocco. Photo courtesy of Prof. Mohamed Taiebine. From the personal collections of the authors.

This cumulative intellectual synthesis reached a structural zenith with the work of Ibn Khaldun. By integrating the preceding centuries of philosophical, geographical, and ethical inquiry, Ibn Khaldun transformed these disparate threads into the first cohesive theory of sociology in his al-Muqaddima (Figure 2) [10].

First documented medical degree at al-Qarawiyyin

**Figure 3:**
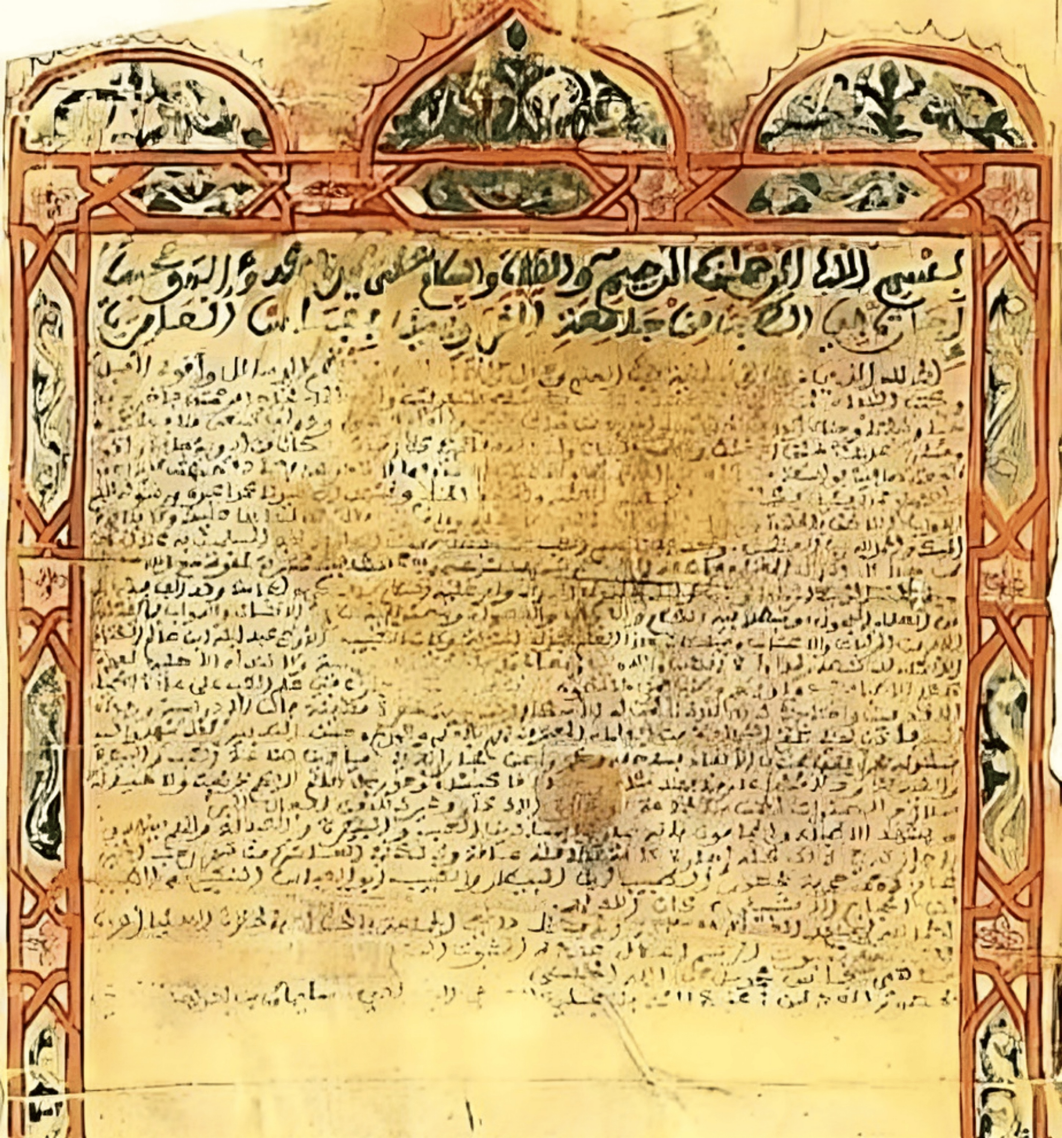
Photo of the original medical degree (ijazah) conferred by the University of al-Qarawiyyin in Fez, Morocco, dating to 1207 CE (603 AH) (courtesy of Prof. Najoua Ghani). From the personal collections of the authors. AH, After Hijra; AD, Anno Domini

The University of al-Qarawiyyin is distinguished by its contributions to medical education. One of the earliest documented medical degrees was conferred in 1207 CE (603 AH) on Abdellah Ben Salah Al Koutami (Figure 3) [3,11]. The historical development of medical certification in the Islamic world established critical precedents for institutionalized academic and professional standards. This degree, which awarded Al Koutami an *Ijaza *(medical license or certificate), is regarded as one of the earliest formalized medical degrees in history, predating many European examples, including the later Latin Licentia [12]. *Ijaza *represented a significant academic accomplishment, enabling Al Koutami to teach and practice medicine, a system that subsequently influenced the development of medical degrees in Europe [12].

Abdellah Ben Salah Al Koutami [3] was mentored by several eminent scientists, including the renowned botanists and physicians Abul Abbas al-Nabati, Abu Mohammed Ibn al-Baytar, and al-Ishbili Ibn al-Hajjaj. His medical certificate authorized him to practice human medicine, veterinary medicine, and pharmacy. This degree was awarded following a rigorous evaluation of his expertise by qualified medical authorities, the judge of Fez (Mohammed Ibn Abdullah Taher), and official witnesses.

In addition to detailing his clinical qualifications, the historical text of his *Ijazah *(Figure 3) provided specific biographical insights into his character and background. The manuscript portrayed Al Koutami as an honest and benevolent individual with a commendable reputation, explicitly noting the absence of any criminal or disloyal conduct in his history. Furthermore, the certificate attests to his religious devotion, describing him as a practicing Muslim who consistently observed his five daily prayers. His medical practice was firmly rooted in the scientific advancements of his time, with his licensing reflecting a comprehensive curriculum that included plant and herbal sciences, pharmacology, and established treatments such as cupping therapy (hijama).

Broadly speaking, the medical curriculum at al-Qarawiyyin was heavily influenced by the works of eminent physicians such as Hippocrates, Galen, Avicenna (Ibn Sina), and al-Razi (Rhazes). Medical students received training in a broad array of subjects, including anatomy, pharmacology, surgery, and ophthalmology [6,7,11]. The influence of Islamic scholars, such as al-Zahrawi, who was considered the father of modern surgery, was evident in the curriculum at al-Qarawiyyin. His contributions to surgical techniques and instruments significantly impacted medical practice in both the Islamic world and Europe.

Influence of Islamic medicine on European universities

The medical knowledge developed and refined at al-Qarawiyyin and other Islamic institutions profoundly influenced European medicine during the Middle Ages during the Middle Ages. As Arabic texts were translated into Latin during the 12th and 13th centuries, European scholars gained access to the medical theories of Avicenna, al-Razi, and al-Zahrawi [13-15]. European universities, particularly those in Salerno, Montpellier, and Bologna, integrated these texts into their medical curricula, and many surgical techniques and diagnostic methods outlined in these texts were adopted by European physicians. By 1241, the curriculum at the Salerno Medical School featured the works of Hippocrates, Galen, Phylaretus, Aristotle, and Theophilus [16], whereas the curriculum at the Bologna Medical School was centered primarily on the teachings of Avicenna [17]. For instance, the Faculty of Medicine at Montpellier was significantly influenced by the Arabic medical tradition. The study of Ibn Sina’s *Canon of Medicine* and al-Zahrawi’s *Kitab al-Tasrif* became central to medical education in Europe [18,19]. These texts provided a foundation for understanding diseases, treatments, and surgical procedures, which continued to shape European medicine until the Renaissance.

Women's leadership in medical education

Fatima al-Fihriya’s establishment of the University of al-Qarawiyyin serves as a foundational model for women’s leadership in education and philanthropy, illustrating the critical intersection between social perception and institutional progress. While contemporary discussions often focus on the complexities of women's roles in Muslim societies, al-Fihriya’s legacy stands as a persistent testament to the transformative potential of female-led intellectual stewardship [4]. This precedent created a fertile environment for women to navigate specialized fields, most notably the healing arts, where historical trajectories diverged sharply between Eastern and Western societal structures [11].

In the Eastern tradition, particularly during the Islamic Golden Age, gender-segregated spaces paradoxically secured professional authority for women. Unlike the Western narrative, which often emphasizes the struggle for institutional re-entry, the East fostered a robust tradition of female healers, midwives, and pharmacologists [20,21]. Because medical practice was deeply linked to the *female gaze - *a requirement of modesty and religious law - women were frequently entrusted with the administration of health spaces, blending spiritual wellness with clinical expertise as a natural extension of their social responsibilities [22].

Conversely, the Western medical narrative followed a path of early integration, succeeded by systemic institutional exclusion. Although figures like Trotula of Salerno demonstrate that women once held recognized teaching roles in early medieval medical instruction, the subsequent formalization of university-sanctioned degrees in the late Middle Ages largely pushed women out of the official sphere [23,24,25]. This necessitated a rigorous, centuries-long battle for re-entry, culminating in 19th-century movements led by pioneers like Elizabeth Blackwell and Elizabeth Garrett Anderson [26]. Consequently, while the presence of women in Western medicine became a hard-won symbol of civil rights and intellectual equality, it remained a functional, albeit shielded, cultural necessity in the East [27].

Re-evaluating the institutional context of al-Qarawiyyin

A crucial distinction in this historical analysis is the institutional architecture of the University of al-Qarawiyyin. While it successfully integrated medicine into its broader pedagogical curriculum, historical evidence supported the existence of a dedicated, formally affiliated teaching hospital (Bimaristan) within its structure [28]. During the Islamic Golden Age, clinical training was primarily conducted through apprenticeship within autonomous Bimaristans - independent healthcare facilities that functioned separately from academic centers [28,29]. The term Bimaristan is derived from the Persian words *Bimar*, meaning disease, and *Stan*, meaning place, essentially referring to a location for the sick [30]. Considered one of the most significant and innovative institutional accomplishments of Islamicate society, the inaugural Bimaristan was founded in Baghdad during the rule of Harun al-Rashid (r. 786-809). These hospitals were not merely centers for patient care; they also functioned as medical schools where future *Hakims* and *Tubibs* received formal training [31]. The establishment of the first dedicated Bimaristan in Fez is most notably linked to the Merinid dynasty, particularly during the late 13th century under the patronage of Sultan Abu Yusuf Yaqub. While the University of al-Qarawiyyin had long functioned as a center for theoretical medical education, the founding of the Sidi Frej Bimaristan [32] around 1286 CE signified the city's shift towards specialized, autonomous clinical institutions. This facility was strategically situated near the Henna Souk to serve the public and gained recognition for its innovative healthcare approaches, notably its pioneering use of music therapy and specialized wards for individuals with mental disorders. Unlike the integrated university-hospital models prevalent in modern medicine, the Sidi Frej Bimaristan operated as a distinct entity, supported by religious endowments (waqf), which enabled it to offer free services and maintain a separate administrative identity from the adjacent mosque-university complex for centuries [31,32]. Today, the convergence of global medical standards is bridging these historical divides, forcing a reassessment of how culture dictates the visibility of female expertise. While the Western model eventually succeeded in standardizing paths for women through legislative reform, it historically overlooked the holistic, community-integrated approaches that characterized Eastern female practitioners for centuries. By recognizing both the institutional triumphs of the West and the persistent, culturally embedded roles of the East, one gains a more comprehensive understanding of how women have acted as architects of human well-being, effectively bridging the gap between historical tradition and modern scientific advancement.

Legacy and key lessons for contemporary medical education

The legacy of al-Qarawiyyin is evident not only in its intellectual achievements but also in its enduring impact on the educational systems of the Islamic world and Europe [3]. The university’s role in fostering a spirit of inquiry, critical thinking, and intellectual exchange laid the groundwork for the development of modern universities and medical education [9,15]. Furthermore, the influence of women on the establishment of such institutions underscores the importance of gender equality in educational and intellectual progress [22,23]. Today, while the Islamic world does not contribute significantly to global scientific research, the historical legacy of institutions like al-Qarawiyyin offers valuable lessons on the importance of investing in education, innovation, and international collaboration. Such a rich heritage should guide Arab countries from the North-African and Middle-East regions to work towards reviving their contributions to global scientific and medical advancement [33-35].

Limitations 

This review acknowledges several methodological constraints inherent to the scope of this historical synthesis. This analysis is based primarily on reliance on published secondary sources, which partially limits the ability to provide new primary evidence regarding the specific transmission of academic administrative practices. Furthermore, the absence of systematic search methods across the full corpus of medieval manuscripts means that this overview remains exploratory rather than exhaustive. Finally, it must be noted that some causal claims regarding the direct influence of Islamic educational structures on later European academic frameworks are interpretative rather than demonstrable in a strict empirical sense, given the complex and fragmented nature of the historical record during this era, while the historical evidences are considered to be trustworthy and reliable.

While Ijaza's credentials reflect a rigorous mastery of pharmacology and clinical theory, current scholarship indicates that there was no anatomical dissection at the University of al-Qarawiyyin due to prevailing religious and cultural norms of the era, contrasting with the later re-emergence of dissection in select European medical schools [29,30]. Consequently, further critical investigation into the pedagogical limitations and methodologies of this institution would further enrich the manuscript by delineating the distinction between its theoretical training and experimental practice.

## Conclusions

In conclusion, the University of al-Qarawiyyin exemplified the intellectual vibrancy of the Islamic Golden Age and its profound influence on global academic education. Its rigorous certification system and diverse multidisciplinary curriculum shaped the modern university model and facilitated a transcontinental exchange of knowledge that spurred the European Renaissance. Historical records of early medical degrees, such as the ijaza awarded to Abdellah Ben Salah Al Koutami, demonstrate a level of institutionalization that bridged the gap between ancient practices and modern professional standards. Moreover, the establishment of al-Qarawiyyin through the philanthropy of Fatima al-Fihriya served as a crucial historical corrective, highlighting women’s significant role in developing educational infrastructure. Although the contemporary scientific output of Morocco has evolved throughout centuries, the legacy of al-Qarawiyyin provided a blueprint for reviving intellectual excellence and leadership in medical education.
